# Sound Effects on Physiological State and Behavior of Drivers in a Highway Tunnel

**DOI:** 10.3389/fpsyg.2021.693005

**Published:** 2021-06-23

**Authors:** Yanqun Yang, Yang Feng, Said M. Easa, Xiujing Yang, Jiang Liu, Wei Lin

**Affiliations:** ^1^College of Civil Engineering, Fuzhou University, Fuzhou, China; ^2^Department of Civil Engineering, Ryerson University, Toronto, ON, Canada; ^3^School of Architecture and Urban-Rural Planning, Fuzhou University, Fuzhou, China; ^4^Department of Civil and Architectural Engineering and Construction Management, University of Cincinnati, Cincinnati, OH, United States

**Keywords:** sound effect, driving behavior, physiological state, heart rate variability, electroencephalography

## Abstract

Driving behavior in a highway tunnel could be affected by external environmental factors like light, traffic flow, and acoustic environments, significantly when these factors suddenly change at the moment before and after entering a tunnel. It will cause tremendous physiological pressure on drivers because of the reduction of information and the narrow environment. The risks in driving behavior will increase, making drivers more vulnerable than driving on the regular highways. This research focuses on the usually neglected acoustic environment and its effect on drivers' physiological state and driving behavior. Based on the SIMLAB driving simulation platform of a highway tunnel, 45 drivers participated in the experiment. Five different sound scenarios were tested: original highway tunnel sound and a mix of it with four other sounds (slow music, fast music, voice prompt, and siren, respectively). The subjects' physiological state and driving behavior data were collected through heart rate variability (HRV) and electroencephalography (EEG). Also, vehicle operational data, including vehicle speed, steering wheel angle, brake pedal depth, and accelerator pedal depth, were collected. The results indicated that different sound scenarios in the highway tunnel showed significant differences in vehicle speed (*p* = 0.000, η^2^ = 0.167) and steering wheel angle (*p* = 0.007, η^2^ = 0.126). At the same time, they had no significant difference in HRV and EEG indicators. According to the results, slow music was the best kind of sound related to driving comfort, while the siren sound produced the strongest driver reaction in terms of mental alertness and stress level. The voice-prompt sound most likely caused driver fatigue and overload, but it was the most effective sound affecting safety. The subjective opinion of the drivers indicated that the best sound scenario for the overall experience was slow music (63%), followed by fast music (21%), original highway tunnel sound environment (13%), and voice-prompt sound (3%). The findings of this study will be valuable in improving acoustic environment quality and driving safety in highway tunnels.

## Introduction

The highway tunnel is a semi-concealed structure with an abrupt change in the external environment (e.g., lighting and sound), usually built on complex terrain to effectively use mountainous areas and protect the natural environment. Driving through a tunnel is a challenging and risky task for drivers. When driving through a tunnel, drivers need to process lots of information within a short time, and their sensory systems should constantly monitor and immediately respond to many environmental variables, leading to increased workload. Besides, the psychological depression generated by the relatively confined space on drivers could result in irritability and tension (Calvi et al., 2012; Feng and Chen, 2017). Under this stressful situation, drivers are more prone to dangerous and risky driving (Yan et al., 2014), resulting in improper behaviors.

It is reported that 60.1% of driving accidents were caused by the improper behavior of the drivers (Wang et al., 2017). Furthermore, driver behavior in tunnels has increasingly become of concern to promote safety (Calvi and D'amico, 2013). Considerable physiological studies have been conducted concerning three types of indicators: heart rate variability (HRV) indicators, electroencephalogram (EEG) indicators, and vehicle indicators. Typically, HRV is used to detect driver workload (Bortkiewicz et al., 2016), sleep stage (Yamakawa et al., 2017), and driving errors that occur in actual driving tasks (Michail et al., 2008). EEG is used to study driver anger (Ping et al., 2014), fatigue (Jap et al., 2009), and alertness level (Kiymik et al., 2004). After these indicators are determined, they could be measured using some exogenous variables to evaluate driving behavior further. Such variables include vehicle speed corresponding to accident rate (Aarts and Schagen, 2006), steering wheel angle determining whether fatigue driving exists (He et al., 2011), and accelerator and brake pedal depth reflecting a driver's speed control and concentration (Caliendo et al., 2013).

Concerning highway tunnels, the contributing factors to driving behavior focus on traffic environment characteristics, such as light (Song et al., 2018), sound (Akamatsu et al., 2003), and alignment (Rudin-Brown et al., 2013). Especially for the acoustic environment, some researchers demonstrate that music could relieve pressure and aggression in drivers (Dalton and Behm, 2007), thus influencing driving behaviors. Specifically, joyful music can distract attention and reduce speed, while sad music can improve lane-keeping ability (Pêcher et al., 2009), and fast-paced music could easily cause speeding (Brodsky, 2001). Some researchers claimed that natural sounds could enhance driving ability (Febriandirza and Chaozhong, 2017). However, the noise will affect the accuracy (Hartley and Williams, 1977) and reduce the alertness level (Smith, 1988) of the driver. In particular, the noise inside a tunnel is increased drastically at the exit due to the sudden connection of the restricted space and the natural environment's open space (Takagi et al., 2000). There are calls for some solutions to alleviate the influence of noise and improve the acoustic environment to improve highway tunnel safety. Some attempts using sound systems have been made to influence drivers when they are driving in tunnels (Guiyangnet, 2015).

This paper aims to identify the effect of the acoustic environment on the physiological state and behavior of drivers in highway tunnels using a scenario-based approach. Five sound scenarios are set in the driving simulation experiment. All objective parameters (HRV, EEG, and vehicle parameters) that might reflect the mental state and subjective judgment of the driver, were analyzed and compared to reveal how driving behaviors vary according to different sounds. The purpose of the study was to provide a practical reference for improving the driving safety level in highway tunnels through the control and management of the acoustic environment.

## Materials and Methods

### Experiment Design

#### Experimental Scenarios

**Figure 1:**
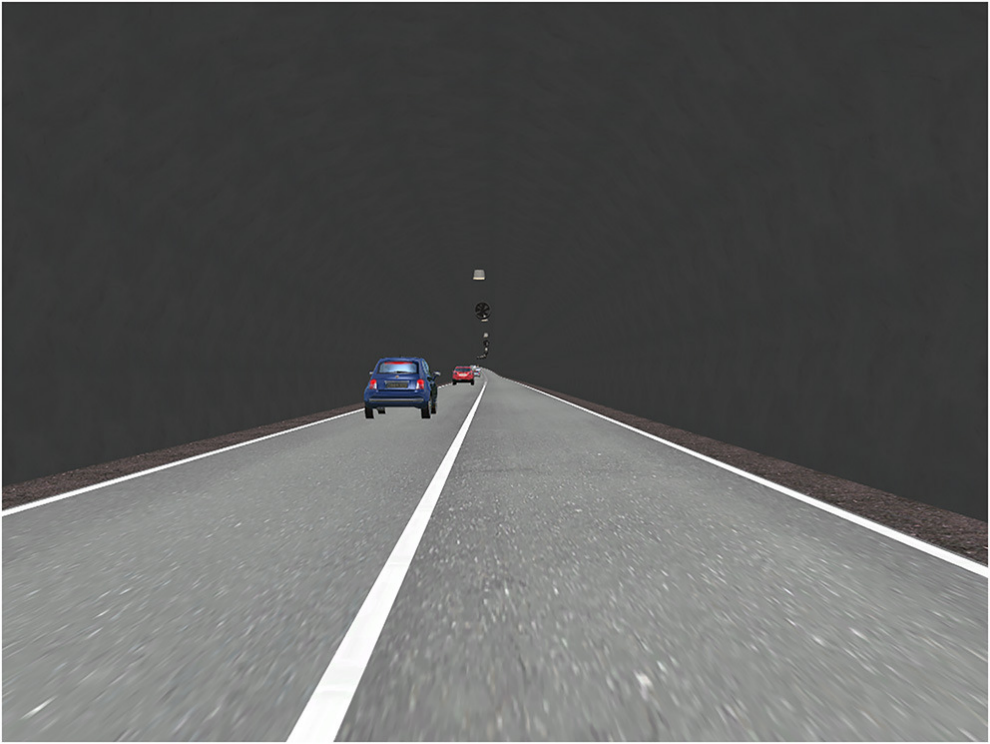
Experimental scenarios of the study highway tunnel.

The experimental road scenario is specific to one direction of a two-way four-lane highway tunnel: one-way two-lane single-hole, as shown in Figure 1. The lane width is 3.75 m and the speed limit is 80 km/h. The study section's total length is 5100 m, where the length from the starting point to the tunnel entrance is 1000 m, the length of the tunnel is 4000 m, and the distance from the tunnel exit to the endpoint is 100 m. The traffic flow in the experimental scenario was randomly generated using passenger cars. The total traffic volume on all lanes was 400 passenger car units per hour. The traffic was not evenly distributed on the lanes. The cars' speed ranged from 80 km/h to 100 km/h. The road alignment is a s-shaped curve with large radius (R = 2000 m), with a longitudinal slope of 2.9%, and horizontal slope of 2%. There is no central reserve set.

The scenario demonstrates the same sound environment from the actual tunnel called the control sound. Four different sound sources were separately mixed with the original sound to form comparative scenarios: slow music, fast music, siren, and voice prompt. The voice prompt was from a woman's voice saying, “Please turn on the lights, slow down, and no-overtaking” and “Here is an accident blackspot, please turn on the lights and slow down.” The siren sound was from special effects generated from the software. The fast music was “Croatian Rhapsody” with 96 beats per minute (BPM), and the slow music was “Canon” with 72 beats per minute (BPM). Before the vehicle entered the tunnel, the usual road noise was played through the speakers connected to the driving simulator. Once they entered the tunnel, the specific sound scenario was played. The sound pressure level was controlled at 70 dB, which was a volume that was loud enough but did not cause discomfort to the driver. To avoid the influence of irrelevant variables on the independent variables, the traffic flow and lighting conditions in the tunnel in each scenario were the same. The experiment was conducted indoors with lights on, and curtains are drawn to control the environmental brightness.

#### Experimental Apparatus

##### Sim Lab Driving Simulator

The main parts of the Sim lab driving simulator are the driving simulator motion platform, a console, four high-definition projectors, and front and rear curtain walls. This equipment was used to collect the vehicle characteristics, such as the depth of the accelerator pedal and brake pedal, steering wheel angle and speed, and acceleration.

##### ErgoLAB Man–Machine Environment System

The ErgoLAB man–machine environment system consists of ECG wireless sensor, photo plethysmo graphic (PPG) wireless blood volume pulse sensor, and electro dermal activity (EDA) wireless skin sensor. The system uses synchronization technology, such as radio frequency physiological recording, which can record, track, and analyze real-time data, including individual physiological, psychological, and behavioral changes simultaneously or within the same period. Also, it could be used for analyzing the physiological data of the individual's driving behavior. The equipment includes three types of metrics, as follows:

1. ECG wireless sensor metrics: measuring range: −1,500 to +1,500 μV, maximum transmission rate: 500 kbps, noise: 1.6 μV (RMS), sampling rate: 256–4,096 Hz, resolution: 16 bits, and accuracy: up to 0.026 μV.
2. PPG wireless blood volume pulse sensor metrics: measuring range: 25–240 bpm, sampling rate: 32–256 Hz, resolution: 1 bpm, and accuracy: ±3 bpm.
3. EDA wireless skin sensor metrics: measuring range: 0–30 μs, sampling rate: 32 Hz/Channel, and resolution: 0.3 μt.

##### Neuroelectrics Wireless EEG

Neuroelectrics Wireless EEG is a wearable wireless EEG system. The recording data can be wirelessly transmitted to the PC software in real time. It can be used to compare and analyze the brain wave changes of the drivers in a specific situation in real time and then analyze the change in his/her mental state. The metrics are as follows: number of channels: 8, 20, 32, bandwidth: 0–250 Hz, sampling rate: 500 SPS, resolution: 24 bits−0.05 μV, and noise: <1 μV RMS (0–250 Hz).

#### Subjects

This experiment hired 45 drivers with valid driving licenses. The subjects were undergraduate or graduate students selected from the university campus, between 20 and 25 years of age, with normal auditory and vision levels, and similar driving age and driving mileage. Before the formal experiment, the subjects were informed to ensure that they get adequate sleep. They could not drink or do other activities that could affect driving before the experiment. Thus, they were all in good physical condition when participating in the experiment. The time of the experiment was scheduled and informed to the subjects to avoid waiting for too long for their turn and to prevent irritability. Their uncorrected visual acuity or corrected visual acuity of both eyes was above 4.9 in the logarithmic visual acuity chart. Their hearing was certified using the tuning fork vibration test. Besides, to avoid the expectation effect of the experiment, the subjects who understood the purpose of the experiment were excluded. After data screening, 38 sets of valid data from 20 male drivers and 18 female drivers were finally obtained. Table 1 shows the average and SD value of age, driving age, and driving mileage of subjects.

**Table 1:** Information of subjects.

	**Age (yr)**	**Driving age (yr)**	**Driving mileage (km)**
Average	23.3	2.7	13947.4
SD	1.1	1.3	6704.1

#### Experimental Procedures

**Figure 2:**
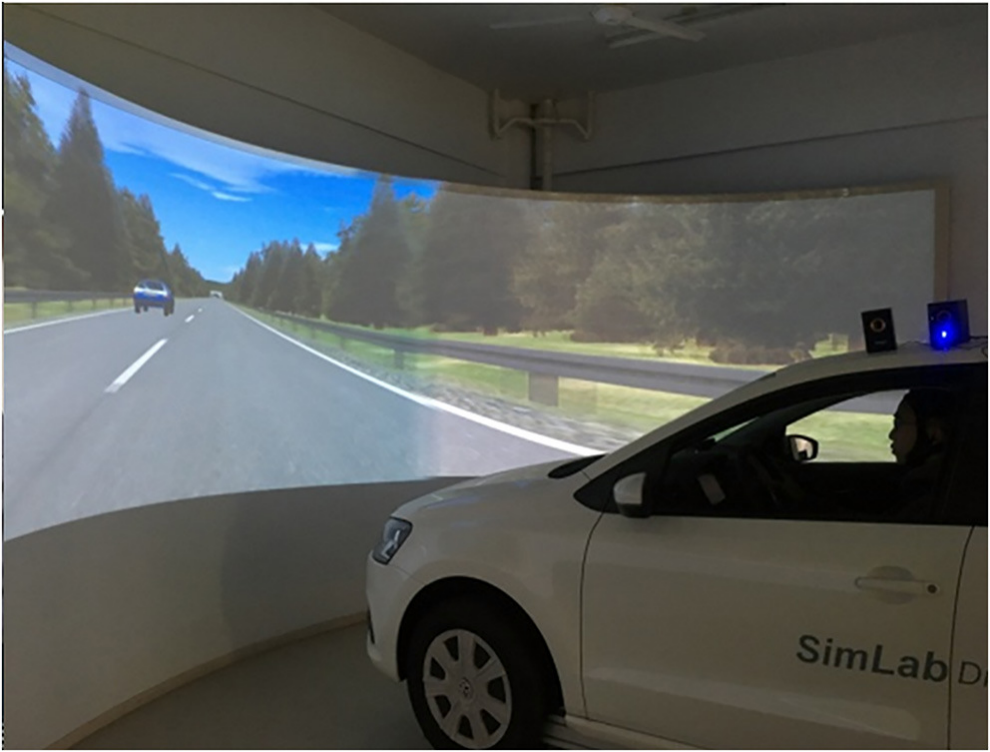
Subject in SimLab driving simulator.

The experimental procedures were as follows:

- To ensure that the equipment was in regular operation, the test experimental road scenario was loaded, and the subjects were allowed to be familiar with the simulator operations without wearing the experimental equipment.
- After the subject was familiar with the simulator, the subject could wear the EEG cap and the physiological collection instrument, as shown in Figure 2. After the instrument was in a stable recording state, the subject could sit in the driving position.
- When the subject was ready, the subject was informed to start the experiment, and the data were recorded at the same time. If the subject had an uncomfortable reaction, the experiment would be stopped and continued after the subject felt normal again.
- The experiment time was controlled to avoid excessive fatigue in the subjects. The driving time for a single scenario was ~4 min. The average time for switching between the scenarios and the instrument's re-adjustment was 1–2 min. The driving scenarios were provided to the subjects in random order to avoid the memory effect (Ali et al., 2020; Onate-Vega et al., 2020; Vollrath et al., 2021). The duration of the entire experiment was 25–30 min.
- After all the scenarios were completed, the participants got off the car and took off the experimental equipment. Then, they were asked to fill in a questionnaire survey based on their experimental experiences.

### Objective Evaluation of the Mental State of the Driver

Based on previous studies, 14 indicators from three categories (HRV index, EEG indicators, and vehicle indicators) were selected to evaluate the sound effects on the mental state of drivers in a highway tunnel, as shown in Table 2.

**Table 2:** Driver's physiological state and driving behavior indicators.

**Indicator type**	**Indicator name**	**Index characteristics**	**Unit**
Heart rate variability indicators (HRV)	AVHR	Average heart rate value, when the heart rate value rises, it can indicate the drivers' tension (Lee et al., 2007).	BPM
	SDNN	Standard deviation of the cardiac interval. It can be used as an indicator of the drivers' nervousness (Miller and Boyle, 2013).	–
	RMSSD	Average value of the difference between adjacent RR intervals. It can be used as an indicator of driving fatigue (Lee et al., 2007).	–
	PNN50	Difference between adjacent R-R intervals is >50 MS as a percentage of the total, which can be used as an indicator of driving fatigue (Lee et al., 2007).	%
	LF/HF	Ratio of low-frequency and high-frequency power. It can be used as an indicator of mental load (Michail et al., 2008).	–
EEG indicators	α-wave (the percentage of α wave to the total energy)	Low-amplitude synchrowave. It is the main waveform recorded in the awake and quiet state. It is generally considered to be related to the brain's preparation activities. This rhythm of brain waves appears when the brain is awake and relaxed (Eoh et al., 2005).	%
	β-wave (the percentage of β wave to the total energy)	High-frequency and low-amplitude asynchronous fast wave. It reflects the alertness of the brain, usually appears when a person's mental state is nervous or excited. When it appears, the brain is prone to fatigue (Ping et al., 2014).	%
	θ-wave (the percentage of θ-wave to the total energy)	Low-to-medium amplitude slow waves. It appears when people turn to sleep from calm and relaxation. It is a manifestation of the central nervous system's inhibited state and is related to working memory load (Lin et al., 2011).	%
	θ/β	When the θ-wave energy increases and the β-wave energy decreases, the ratio increases, which is usually used to characterize drivers' fatigue (Jap et al., 2011).	–
	(θ + α)/β	Composite index of (θ + α)/β energy, which can be used to characterize driving fatigue (Jap et al., 2009).	–
Vehicle indicators (VB)	Vehicle speed	Distance traveled by the car in a unit of time. It can be used to study the emotions of the drivers (Aarts and Schagen, 2006).	km/h
	Steering wheel angle	Angle at which the steering wheel is turned. It can be used to study distractions and drivers' emotions (He et al., 2011).	rad
	Accelerator pedal depth	Depth of the drivers' accelerator pedal. It can be used to study the stability of the drivers (Caliendo et al., 2013).	rad
	Brake pedal depth	Depth of the driver's brake pedal can be used to study the stability of the drivers (Caliendo et al., 2013).	rad

### Subjective Evaluation of Driving Behavior

The subjective feeling of the drivers under different sound scenarios was collected through a questionnaire survey. They were immediately asked to fill in the questionnaire based on their real feelings in the experiment after they finished all the driving scenarios. The questionnaire measures the physiological and psychological feelings of the drivers in four aspects, including driving safety (Q1), driving comfort (Q2), driving load (Q3), and driving fatigue (Q4), using a Likert five-level scale. The main questions of the questionnaire were as follows:

Q1: Please describe your perception of your speed and vehicle spacing in the process of driving under different sound scenarios.
Q2: How comfortable are you when driving under different sound scenarios?
Q3: Please describe the urgency you feel of driving out of the tunnel under different sound scenarios.
Q4: How tired are you when driving under different sound scenarios?
Q5: In your overall driving experience, what is the best one of the five sound scenarios?

### Data Screening

In the driving simulation experiment, the loose earlobe or weak acquisition signal would cause inaccuracy or missing data. Among the 38 sets of HRV data of each scenario, up to 23 sets of incomplete or bad data were excluded from the analysis. The EEG data were first filtered, and then the noise with ICA (independent component analysis) algorithm was removed (Makeig et al., 1996; Lee et al., 1999; Jung et al., 2000). The stability of the signal, number of artifacts, sufficiency of the markers, and mutual signal influence with the heart rate monitor were also considered. Vehicle operational data were obtained from the driving simulation platform. Vehicle operational data of the subjects familiar with the experiment and those of the pre-experiment were removed.

## Results

### Sound Effects on HRV

#### Analysis of HRV Indicator

In terms of the emotional level, a previous study showed that it was positively correlated with emotional level (Mather and Thayer, 2018). The emotions are in the tensest state under the siren sound stimulus, followed by the voice prompt. The emotional level under the fast and slow music scenario is lower than the original sound scenario.

**Figure 3:**
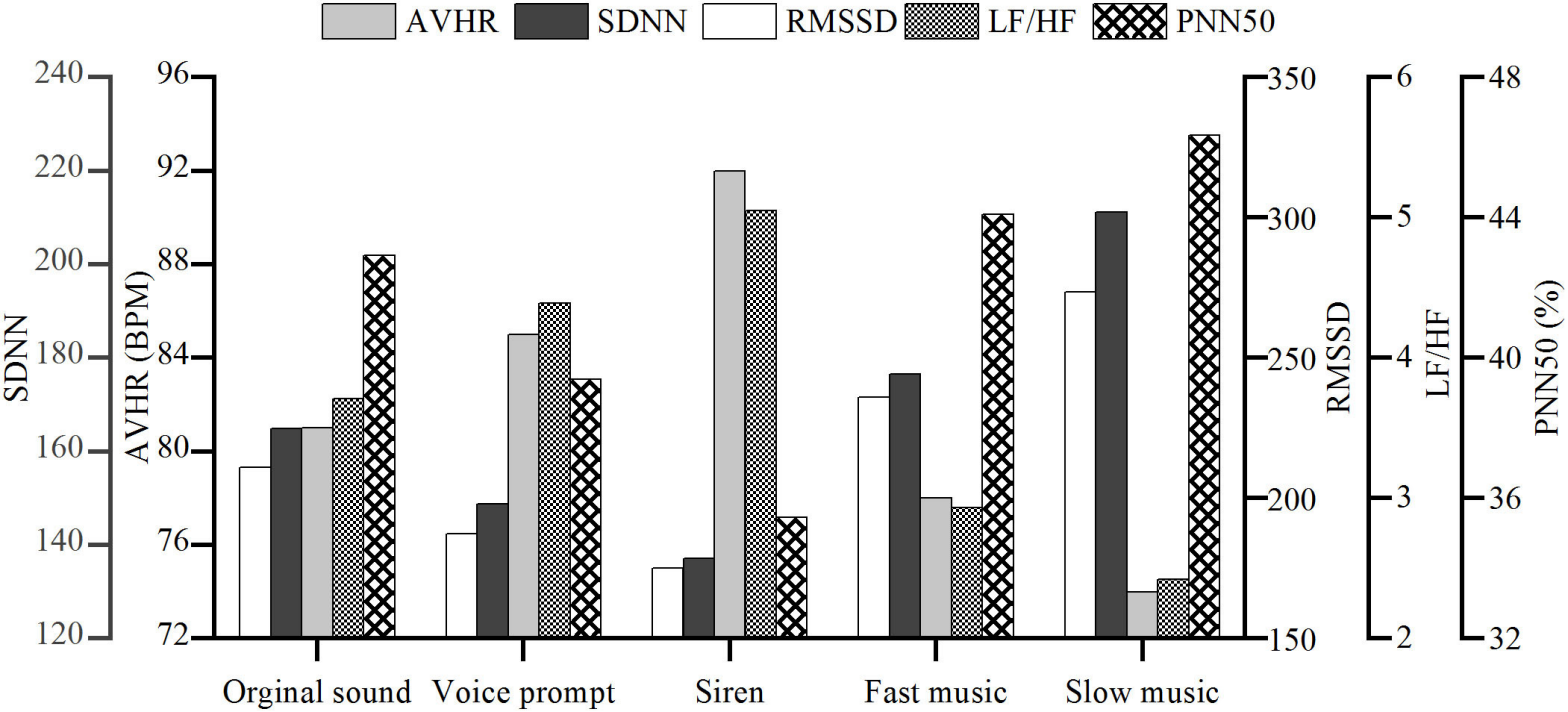
Change of heart rate variability (HRV) indicator for different sound scenarios.

A previous study showed that SDNN, RMSSD, and PNN50 were all reduced when driving fatigue and stress increased (Lee et al., 2007). It is indicated in Figure 3 that AVHR, SDNN, RMSSD, and PNN50 show similar trends under different sound scenarios, all of which reached the lowest level in the siren scenario. Under the slow music scenario, the three types of indicators were at the highest level, and it could be considered that their emotional level and workload as the most stable.

The ratio of LF/HF under different sound scenarios is obviously different, as indicated in Figure 3. According to previous study results, the lower the emotional level, the higher the LF/HF ratio, and the degree of fatigue and workload were negatively correlated with the LF/HF ratio (Michail et al., 2008). This research showed that the LF/HF ratio of the drivers was the lowest under the slow music scenario. The emotional level was stable, followed by fast music, original sound, voice prompt, and siren.

#### Relationship Between Scenarios and HRV

One-way repeated measure ANOVA was used to analyze the effects of different sound scenarios on the HRV. The test results of Mauchly sphericity and the within-subject effect are shown in Table 3 and Table 4. As noted, different sound scenarios have no significant difference on HRV, as indicated by the *p*-value of the significance test (all *p* > 0.05), mainly because of the limited sample size (15).

**Table 3:** Mauchly's test of sphericity of HRV, EEG, and vehicle behavior indicators.

**Variable**	**Mauchly's W**	**Approx. Chi-square**	**df**	**Sig**.	**Epsilon Greenhouse-Geisser**
AVHR	0.514	150.603	9	0.076	0.736
SDNN	0.085	570.619	9	0.000^*^	0.431
RMSSD	0.140	460.094	9	0.000^*^	0.495
PNN50	0.230	340.399	9	0.000^*^	0.552
LF/HF	0.011	56.493	9	0.000^*^	0.369
α-wave	0.646	16.348	9	0.060	0.833
β-wave	0.459	29.159	9	0.001^*^	0.709
θ/β	0.006	191.621	9	0.000^*^	0.341
(θ + α)/β	0.006	188.510	9	0.000^*^	0.336
Vehicle speed	0.392	35.014	9	0.000^*^	0.672
Steering wheel angle	0.053	110.108	9	0.000^*^	0.450
Brake pedal depth	0.155	69.717	9	0.000^*^	0.482
Accelerator pedal depth	0.649	16.201	9	0.063	0.859

*Greenhouse-Geisser correction is used when it does not meet the hypothesis of Mauchly sphericity (p < 0.05)*.

* *p < 0.05*.

**Table 4:** Test results of within-subject effects on HRV, EEG, and vehicle behavior indicators for different sound scenarios.

**Variable**	**Inspection type**	**Type III sum of squares**	**df**	**Mean squares**	***F***	**Sig**.	**Partial eta squared**
AVHR	Assumed sphericity	326.892	4	81.723	0.137	0.968	0.004
SDNN	Greenhouse-Geisser	103547557.8	1.724	60069780.5	0.277	0.726	0.011
RMSSD	Greenhouse-Geisser	108469991.0	1.982	54741080.4	0.215	0.805	0.009
PNN50	Greenhouse-Geisser	527.3	2.209	238.7	0.369	0.714	0.015
LF/HF	Greenhouse-Geisser	129800.8	2.153	60293.8	2.484	0.089	0.090
α-wave	Assumedsphericity	0.005	4	0.001	1.245	0.294	0.033
β-wave	Greenhouse-Geisser	0.046	2.833	0.016	2.433	0.072	0.059
θ/β	Greenhouse-Geisser	119.8	1.365	87.7	1.437	0.244	0.036
(θ+α)/β	Greenhouse-Geisser	161.1	1.342	120.1	1.574	0.219	0.039
Vehicle speed	Greenhouse-Geisser	4588.7	2.687	1708.0	7.844	0.000^*^	0.167
Steering wheel angle	Greenhouse-Geisser	0.000045	1.802	0.000025	5.606	0.007^*^	0.126
Brake pedal depth	Greenhouse-Geisser	0.015	1.927	0.008	3.128	0.051	0.074
Accelerator pedal depth	Assumed sphericity	0.001	4	0.000	1.985	0.099	0.048

* *p < 0.05*.

### Sound Effects on EEG

#### Analysis of EEG Energy

##### α-Wave

**Figure 4:**
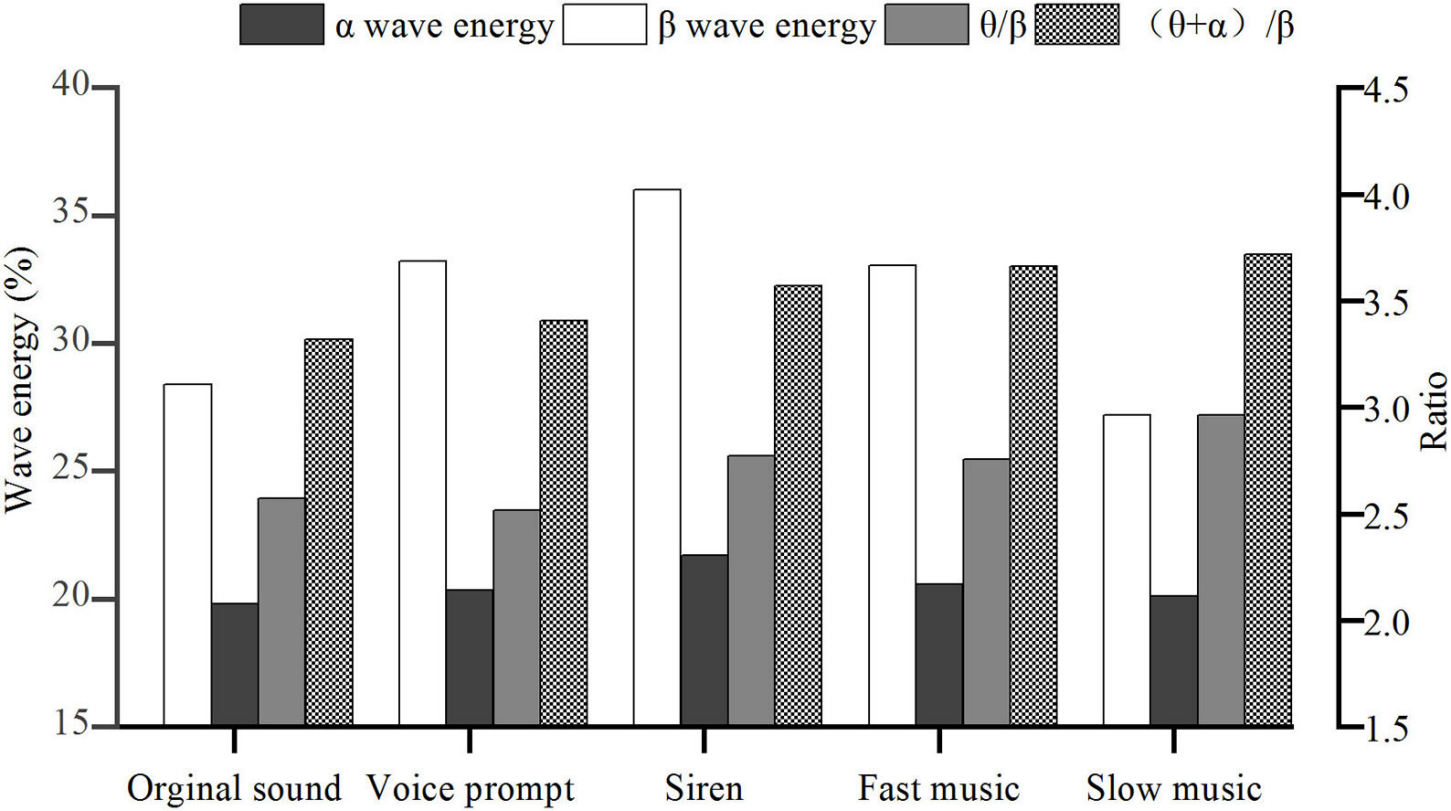
Change of electroencephalogram (EEG) indicators for different sound scenarios.

Regarding α-waves, a previous study showed that they appear when the brain was awake and could be used to measure the soberness degree of the brain (Eoh et al., 2005). The higher the average α-energy, the higher the soberness of the driver. The results in Figure 4 show that:

1. Under the original sound scenario, when the drivers entered the tunnel, the average energy of the α-wave was significantly reduced. Therefore, when driving in the tunnel, it is more likely to cause driving fatigue.
2. Fast and slow music have similar effects compared with the original sound. Under the fast and slow music scenarios, the energy of α-wave was higher than that of other scenarios without music, indicating that music can effectively increase the soberness of the drivers.
3. The comparison shows that the α-waves in drivers under the siren scenario are much higher than the other scenarios. Therefore, although the siren makes the driver feel uncomfortable, it can improve brain soberness, thereby increasing driving safety.

From the above analysis, siren appears to be the most effective sound influencing the α-waves in the drivers when driving inside the tunnel.

##### β-Wave

β-Waves usually appeared when people's mental state was nervous or excited, and the brain was prone to fatigue (Prinzel et al., 1995; Hong et al., 2005). As noted in Figure 4, both original sound and slow music can reduce a driver's mental tension, and the effect of slow music is better than the original sound. Voice prompt, siren, and fast music all increase the nervousness of driving in the tunnel. The siren sound has the most significant effect on β-wave, followed by fast music and voice prompt. When a driver's mental state is nervous and excited, he/she is more prone to dangerous behaviors, such as speeding and overtaking (Gabany et al., 1997; Hennessy and Wiesenthal, 1999; de la Fuente et al., 2017). If the nervous level is increased to a certain extent, it is easy to cause fatigue.

##### θ-Wave and θ/β

When a driver went from a normal state to a fatigued state, the θ-wave significantly increased, and the β-wave decreased significantly, and the θ/β value increased significantly (Kiymik et al., 2004; Ping et al., 2014). The smaller the value, the closer to the normal emotional state. As shown in Figure 4:

(1) Under the voice-prompt scenario, the drivers were sober, and their mental state was stable. This conclusion was mutually confirmed with the α-wave energy.

(2) Under the slow music scenario, the θ/β value in the tunnel was the highest among all scenarios, indicating that this type of music has a specific effect on causing driving fatigue.

##### (θ + α)/β

When the value of (θ + α)/β is large, the alertness and fatigue of the driver were high. As noted in Figure 4, the alertness and fatigue of the driver were at the lowest level under the original sound scenario.

**Figure 5:**
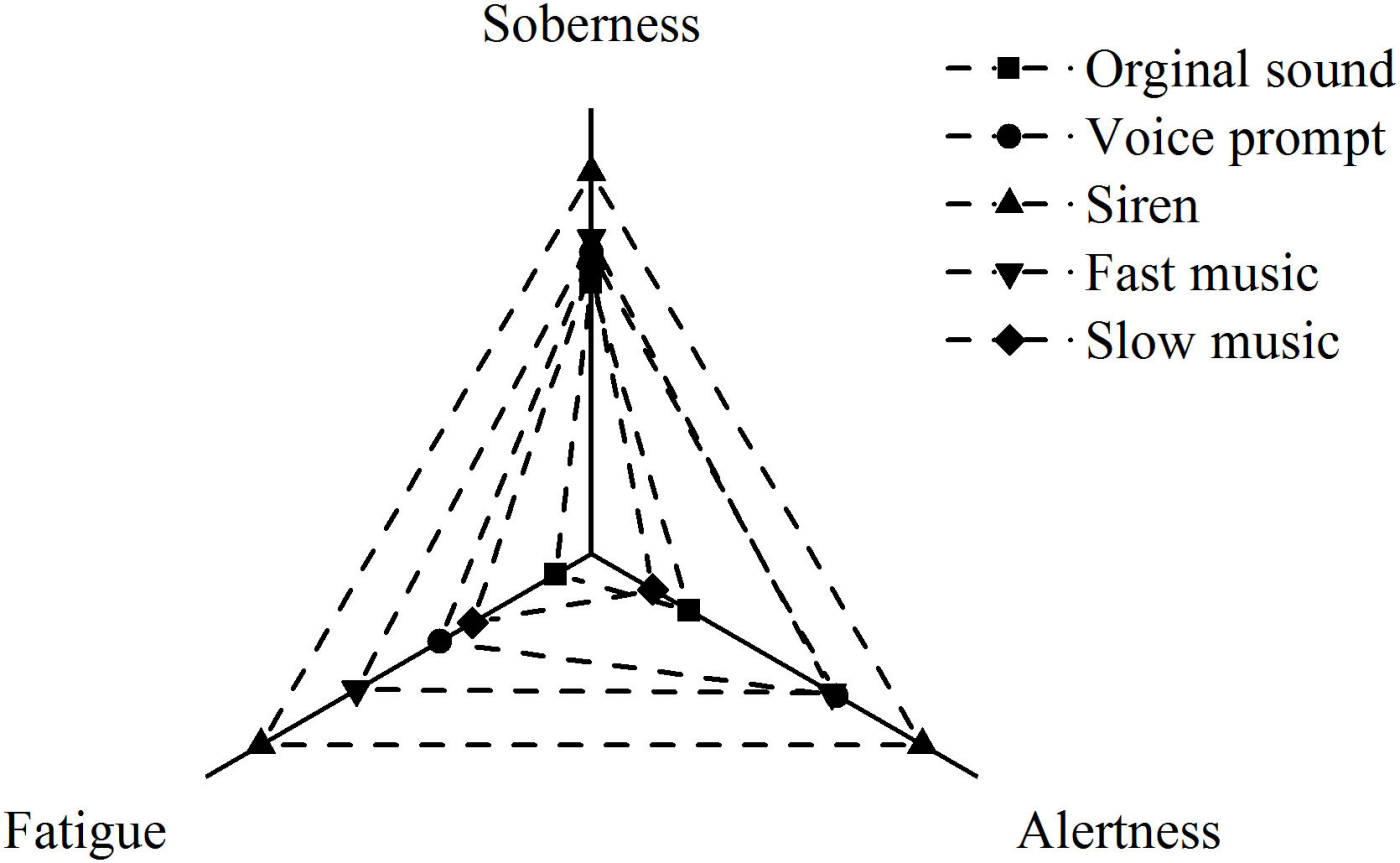
Effect of different sound scenarios on drivers' physiological state considering electroencephalogram (EEG) indicators.

Figure 5 is a schematic diagram of the sound changes in three dimensions based on the three brain wave energies, where the α-wave stands for soberness, the β-wave stands for alertness, and the θ-wave stands for fatigue. As noted, the siren can increase the driver's soberness and arouse the driver's alertness when driving in the tunnel, but it will cause the most driver fatigue. Slow music appears to produce the lowest alertness, although it causes the least fatigue. The driver's alertness of the slow music is lower than that of the original sound. This is possibly due to driver distraction when driving under slow music. However, slow music produces a little more soberness than the original sound. In conclusion, considering only EEG indicators, the siren is the most effective sound for increasing alertness and soberness when driving in the tunnel.

#### Relationship Between Scenarios and EEG

According to the EEG indicators, the significance of the influence of different scenarios was analyzed using the one-way repeated measured ANOVA. According to the test results of Mauchly sphericity and the within-subject effect (Table 3 and Table 4), different scenarios had no significant difference in drivers' EEG (all *p* > 0.05). However, different sound scenarios could possibly affect the β wave (*p* = 0.072, η^2^ = 0.059).

### Sound Effects on Vehicle Behavior

#### Analysis of Vehicle Indicators

Vehicle indicators, including average speed, steering wheel angle, and acceleration and deceleration pedals of the driving vehicle, were selected to analyze different sound scenarios.

##### Vehicle Speed

**Figure 6:**
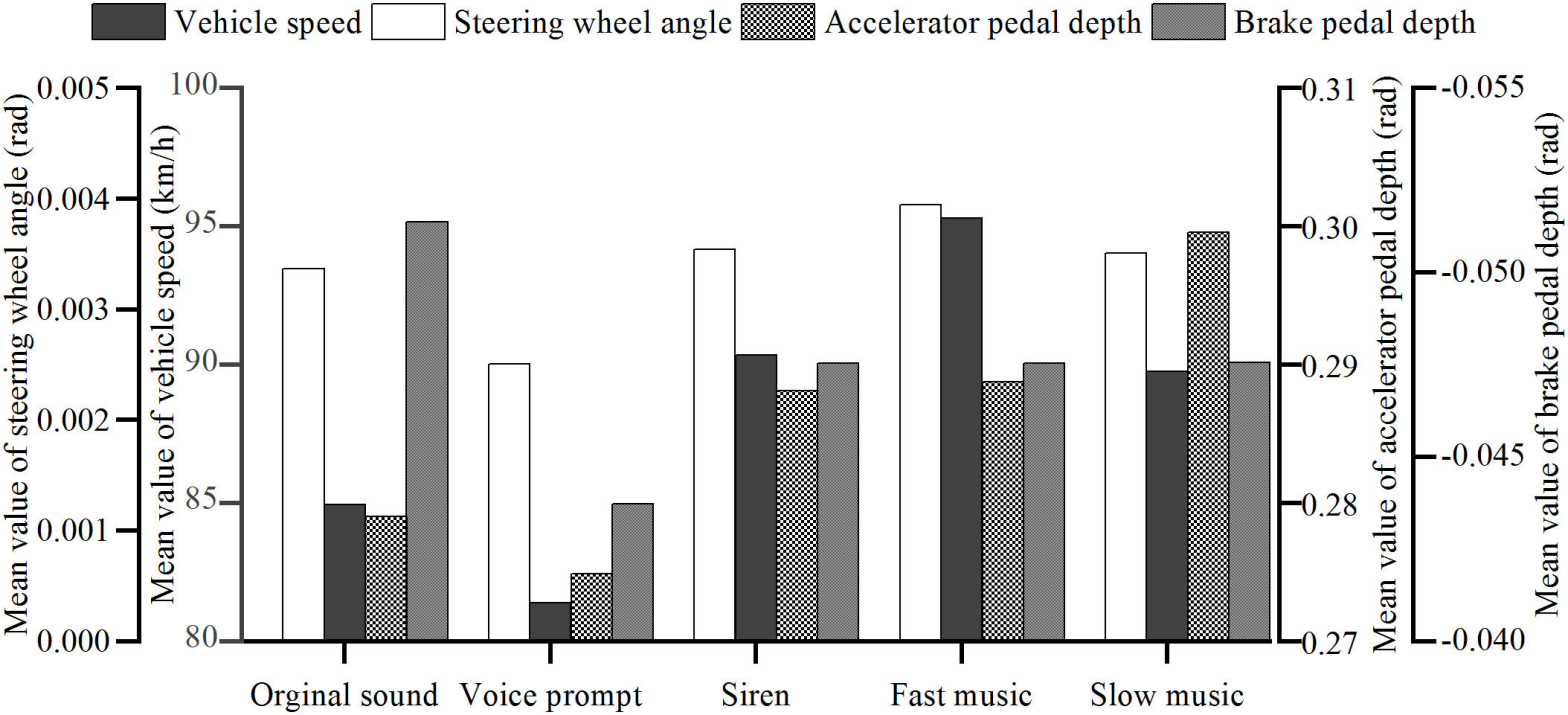
Mean values of vehicle indicators under different sound scenarios.

The driving speed change was the most direct response when a driver was subjected to any stimulus caused by the driving environment. It is indicated in Figure 6 that, under the voice prompt scenario, the drivers could control the vehicle speed most closely to the tunnel speed limit of 80 km/h. The reason might be that the direct command, such as the voice prompt, enabled the driver to control the speed better. The siren, which also served as a warning, has the opposite effect. The siren stimulates the drivers' nervous emotion and increases the vehicle speed. Slow music makes the drivers relax but reduces the alertness of speed control. Fast music is most likely to cause vehicle speeding behavior, consistent with a previous study (Brodsky, 2001).

The overall vehicle speeds from top to bottom are fast music, siren, slow music, original sound, and voice prompt. In terms of speed change, the largest one is fast music, and the smallest one is slow music. These results indicate that slow music positively affects the smooth increase of the overall driving speed, where the drivers have the largest acceleration under fast music, which is not good for safety. For voice prompt, although the overall speed was the closest to the speed limit, the standard deviation was the largest. The reason could be that the drivers need to accelerate and frequently decelerate to correct the speed as the response to the stimulation caused by the voice prompt. These frequent operations can easily lead to distraction and fatigue in driving.

##### Steering Wheel Angle

From the analysis in Figure 6, the average value of the vehicle's steering wheel angle under the voice-prompt environment was at the lowest level in all different sound scenarios. This was due to the clear “no-overtaking” voice message in the voice prompt, making the driver pay more attention to the steering wheel's control. However, frequent operations lead to a larger deviation of steering wheel angle. The effect of slow music on the steering wheel's control shows no significant difference than the original sound scenario, so nor the siren sound. The fast music scenario shows that the average steering wheel angle is larger, indicating that the driver's mood is relatively high in this environment, and dangerous behaviors, such as overtaking and speeding might occur.

##### Acceleration and Deceleration Pedal Depth

Compared with the control scenario, the average values of acceleration and deceleration under the voice-prompt scenario were reduced (Figure 6), indicating that the drivers paid more attention to the pedal control under a clear voice prompt. The drivers' better pedal control under the voice-prompt sound is good for driving security in the tunnel.

#### Relationship Between Scenarios and Vehicle Behavior

The significance of scenarios was analyzed using the one-way repeated measure ANOVA. According to the test results of Mauchly sphericity and the within-subject effect (Table 3 and Table 4) different sound scenarios show significant effect on vehicle speed (*p* = 0.000, η^2^ = 0.167) and steering wheel angle (*p* = 0.007, η^2^ = 0.126). Besides, the impact on brake pedal depth is quite close to the significance level (*p* = 0.051, η^2^ = 0.074).

### Sound Effects on Driving Behavior Scores

**Figure 7:**
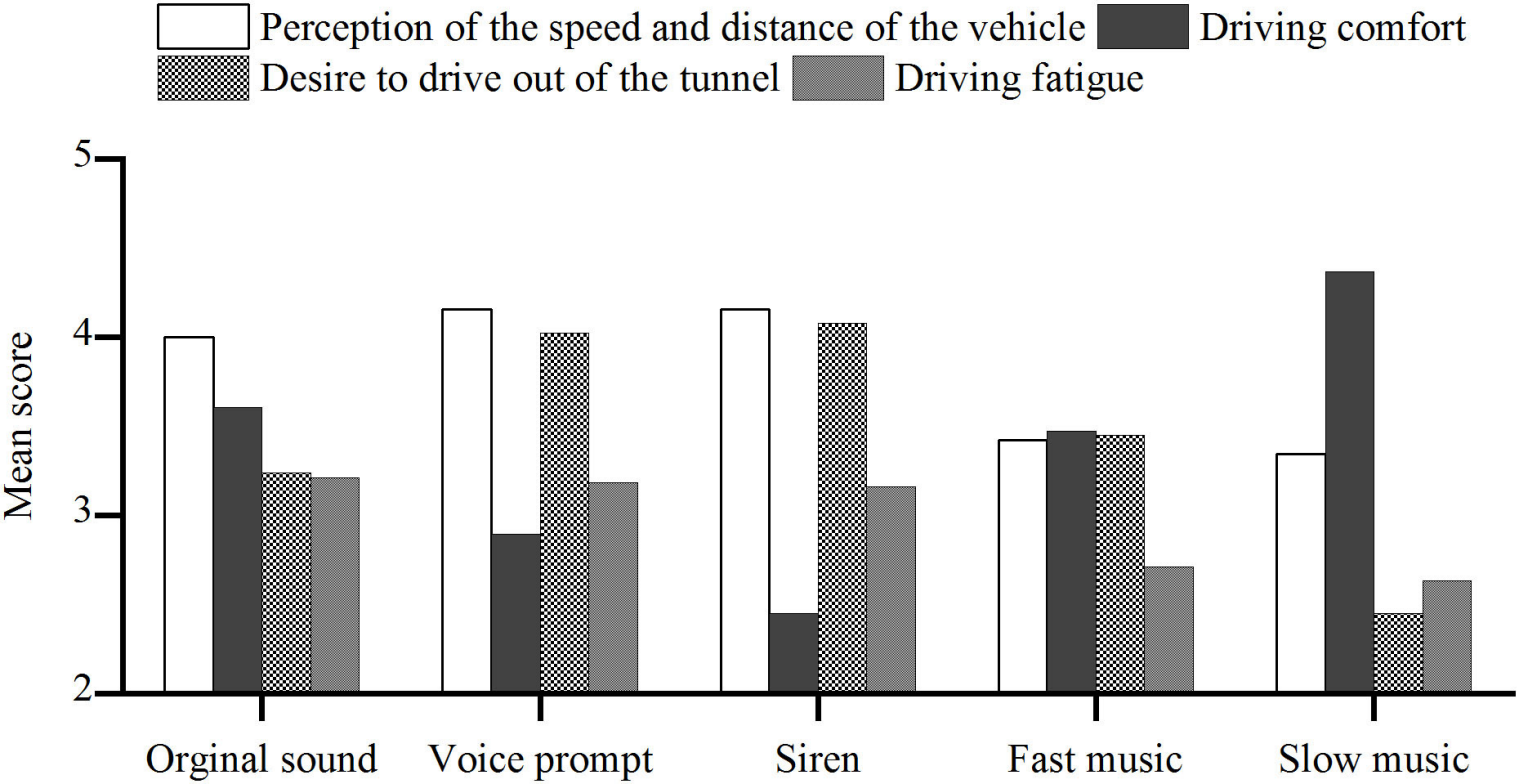
Mean values of the subjective opinions on driving behavior under different sound scenarios.

The results shown in Figure 7 indicate that drivers had a better perception of driving safety under three sound scenarios (original sound, voice prompt, and siren). Among them, the effects from the siren and voice-prompt sounds were more than those of other sounds. The siren sound stimulated the drivers to be alert, and the voice prompt reminded the drivers to pay attention to speed. The speed and distance perception values are the lowest when drivers are driving under fast and slow music. Compared to the original sound, both kinds of music can weaken the driver's alertness, thereby weakening vehicle-driving safety. The siren and voice-prompt sounds are better than the original sound in arousing alertness.

Driving comfort is different under the five-sound scenarios. The siren brings the most uncomfortable experience to the driver. The voice prompt decreases the driver's comfort but increases the drivers' safety performance at the same time. Compared with the original sound of the tunnel, the slow music causes a little more comfortable feeling. For the fast music, the driving comfort might be greatly improved, but at the same time, driving safety might be reduced.

The siren and voice-prompt sounds significantly increase the driver's workload with their high-frequency tones and information volume so that the drivers have a stronger desire to drive out of the tunnel as soon as possible. Compared with the original sound scenario, workload increases slightly under the fast music scenario. Slow music seems to produce the lowest workload.

Under the original sound scenario, the noise composed of natural wind and machine sounds makes the driver feel monotonous and boring, causing fatigue. The siren and voice prompt have a specific promotion effect on the slight reduction of fatigue. Both kinds of music can reduce fatigue and enhance the positive stimulus, while the effect of slow music is more substantial than that of fast music.

Besides, most of the subjects (63%) chose slow music as the best background sound in the tunnel, followed by fast music (21%), whereas voice prompt was the least preferred (3%). The selection of slow music as the best sound environment shows that driver's demand for the sound is mainly based on driving comfort and pleasing experience, while safety is a second consideration, which somewhat reflects young people's lifestyle.

## Conclusions

In this paper, the effects of five kinds of sounds on the physiological states and behavior of drivers in a highway tunnel context were studied. A detailed analysis of the drivers' HRV, EEG, vehicle operational data, and questionnaire data obtained from the driving simulation experiment with five different sound scenarios in a tunnel was performed. The following conclusions could be drawn:

The results of HRV indicators showed that slow music had the most effects on increasing driving comfort and reducing driving load, followed by fast music, while voice prompt and siren could generate a negative effect (compared with the original sound). In terms of EEG indicators, inferring from the α-wave energy, all sound scenarios could improve driver's soberness in the tunnel compared with the original sound, with siren having the most effect, followed by voice prompt, fast music, and slow music. For the β-wave energy, it could be inferred that slow music reduced the nervousness than the original sound, but voice prompt, fast music, and siren alarms could increase nervousness. As indicated by the θ/β and according to the (θ + α)/β value, the best sound environment for driving fatigue was voice prompt.

Compared with the original sound, the voice prompt could release fatigue, while the siren, fast music, and slow music produced more fatigue, with slow music having the largest effect. In the schematic diagram based on three brain waves' energy, the siren aroused the highest alertness and soberness levels while it caused enormous fatigue. Slow music would lower the alertness level below driving under the original sound. In terms of the emotional state of the drivers, according to the subjective questionnaire, the best sound was slow music, followed by fast music, siren alarm, voice prompt, where all sounds were better than the original sound. Note that some results are contradictory, e.g., the results of wave radio value and subjective questionnaire. However, the subjective questionnaire results were more reliable, as the indication of different combinations of EEG waves is still being explored. In terms of vehicle indicators, the results showed that the closer to the tunnel speed limit and the smaller the speed SD, the higher the driving safety. Voice prompt was the only sound that could improve the driving safety compared with the original sound. The steering wheel angle, acceleration, and deceleration pedal depth indicators were consistent with the vehicle speed results.

Slow music had the advantage of increasing driving comfort and reducing driving load, making the driver feel better, consistent with the subjective judgment of the driver. At the same time, slow music might distract the driver and draw his attention away from the driving task, thereby weakening driving safety. Fast music might lead to speeding, though it would improve the mental state of the driver. Although the siren showed significant effects on alerting drivers, it is obviously the least preferred sound scenario, and the lasting time and frequency should be carefully controlled to avoid fatigue and discomfort. Voice prompt had the drivers alert on their driving task, but drivers would feel tired following systematic instructions.

Based on the preceding remarks, slow music was the best sound for drivers' pleasant experience when drivers were driving in a highway tunnel, while it had the disadvantage of reducing driving safety. The siren sound could increase drivers' soberness and arouse drivers' alertness when driving in the tunnel, but the drivers will feel tired and uncomfortable if it lasts for a long time (4 min in this study). Therefore, we suggest that slow music could be played while driving inside the tunnel, and when entering and leaving a tunnel, or some emergencies happen, the siren sound could be used to call the driver's attention and focus on the driving task.

This study enriched the research on the effect of different sounds on driver behavior and physiology under a highway tunnel condition. It provided practical references for safety professionals and the formulation of safety rules. However, due to the impact of the COVID-19 epidemic, this study only recruited college students as experimental subjects, and the experiments were conducted using a driving simulator. “Croatian Rhapsody” and “Canon” were chosen as typical “fast music” and “slow music,” respectively, mainly because these two sounds have different BPM, and are well-known worldwide. The other elements of music indeed have different effects on driving (Millet et al., 2019). More experiments should be carried out in the future to study the effects of different elements of music.

## Data Availability Statement

The raw data supporting the conclusions of this article will be made available by the authors, without undue reservation.

## Ethics Statement

The studies involving human participants were reviewed and approved by Ethics Committee of the College of Civil Engineering, Fuzhou University. The patients/participants provided their written informed consent to participate in this study. Written informed consent was obtained from the individual(s) for the publication of any potentially identifiable images or data included in this article.

## Author Contributions

YY and WL: study conception and design. XY and YF: data collection. YF, XY, JL, WL, and SE: analysis and interpretation of the results. YF, YY, XY, SE, JL, and WL drafted the manuscript. All authors reviewed the results and approved the final version of the manuscript.

## Conflict of Interest

The authors declare that the research was conducted in the absence of any commercial or financial relationships that could be construed as a potential conflict of interest.
